# Supplementary material for: DMRforPairs: identifying Differentially Methylated Regions between unique samples using array based methylation profiles
Source: BMC Bioinformatics. 2014 May 15;15:141. doi: 10.1186/1471-2105-15-141 (PMC4046028; doi:10.1186/1471-2105-15-141)
Supplement: Additional file 2 — DMRforPairs output for the comparison of A431-MCF7 and NA17018-NA17105. Please start from the HTML files in each folder. Available via the BMC Bioinformatics website. [file 1471-2105-15-141-S2.zip › 1394847754114233_MOESM2_ESM/A431_MCF7/figures/19871.pdf]

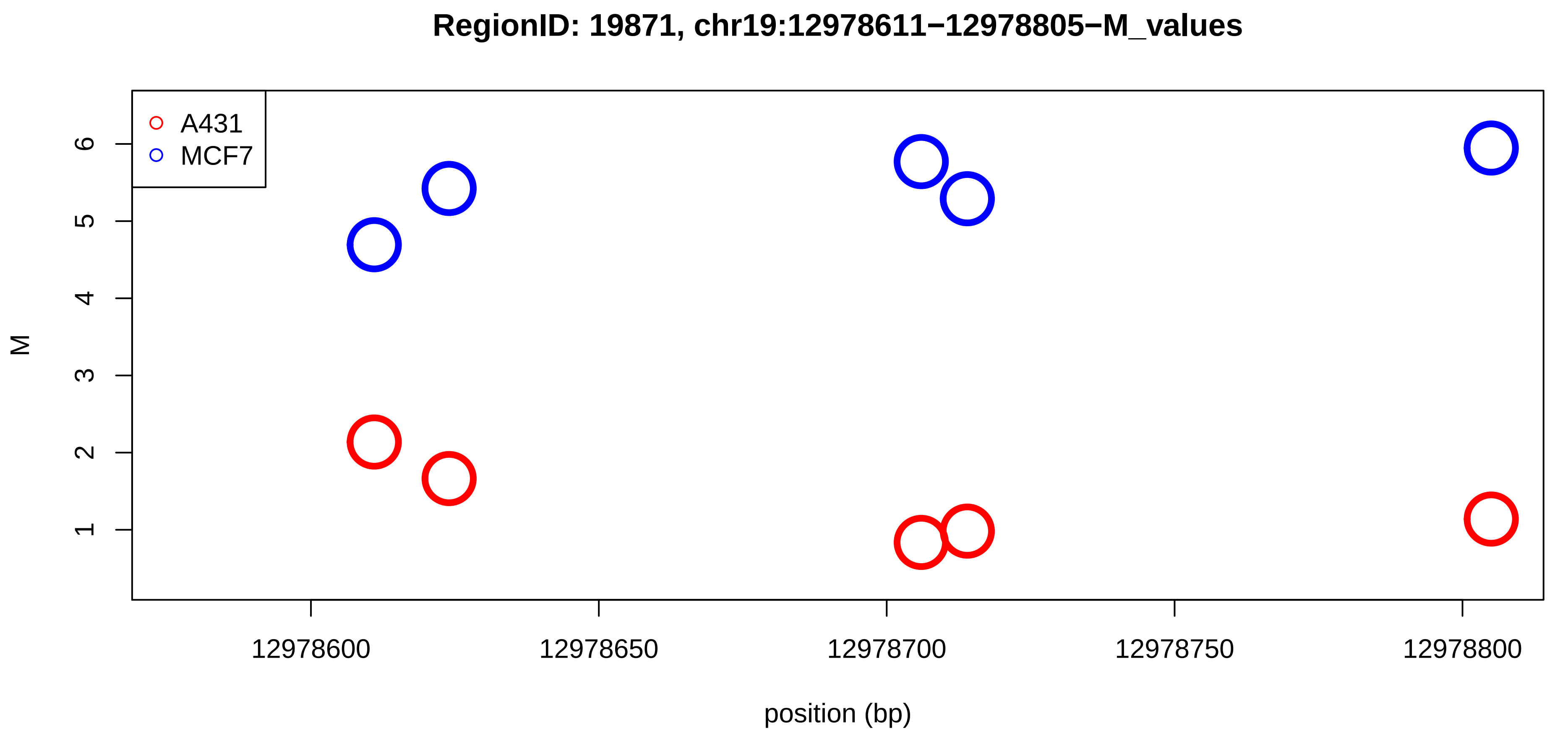
RegionID: 19871, chr19:12978611−12978805−M_values
A431
MCF7
M
position (bp)
12978600
12978650
12978700
12978750
12978800
1
2
3
4
5
6

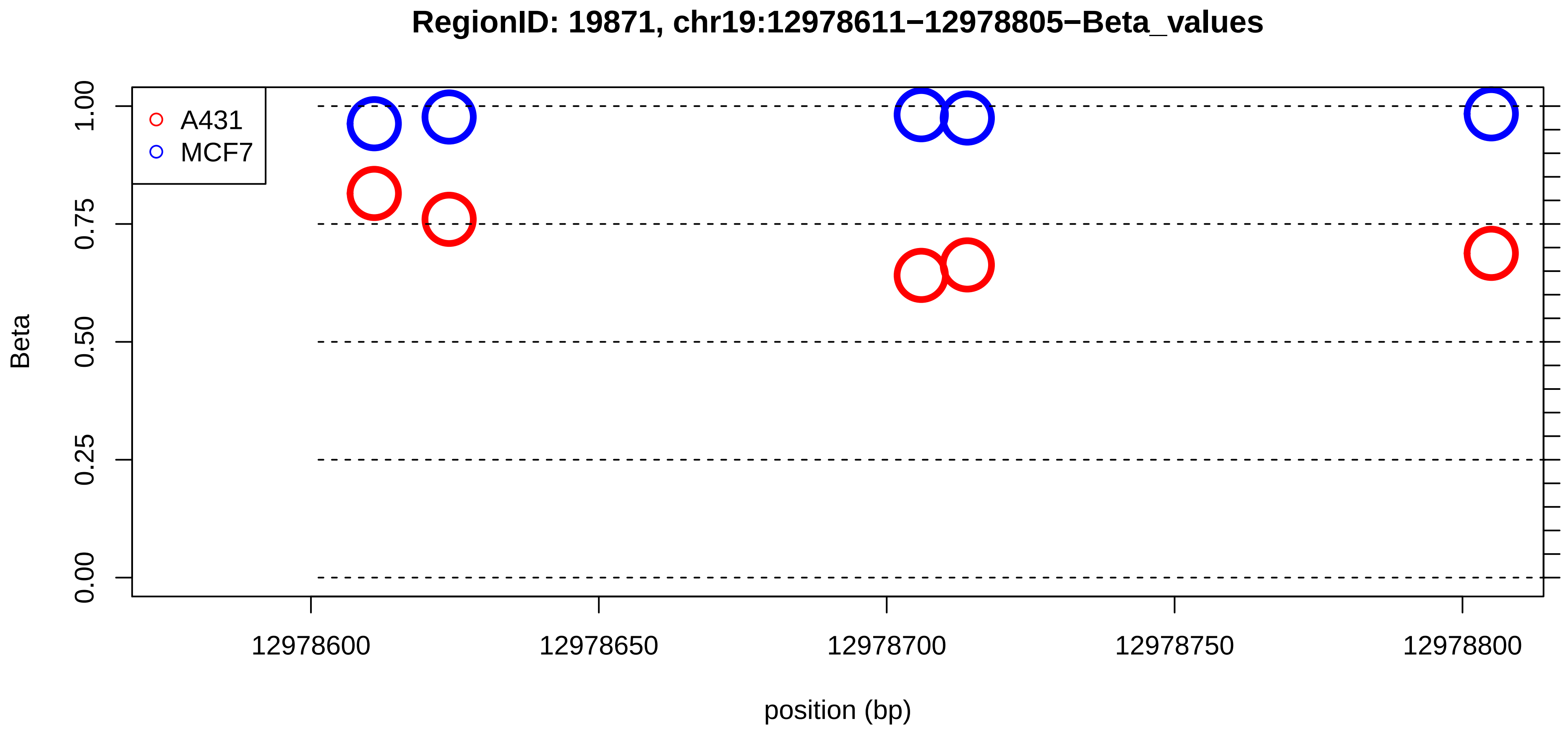
RegionID: 19871, chr19:12978611−12978805−Beta_values
A431
MCF7
Beta
position (bp)
12978600
12978650
12978700
12978750
12978800
0.00
0.25
0.50
0.75
1.00

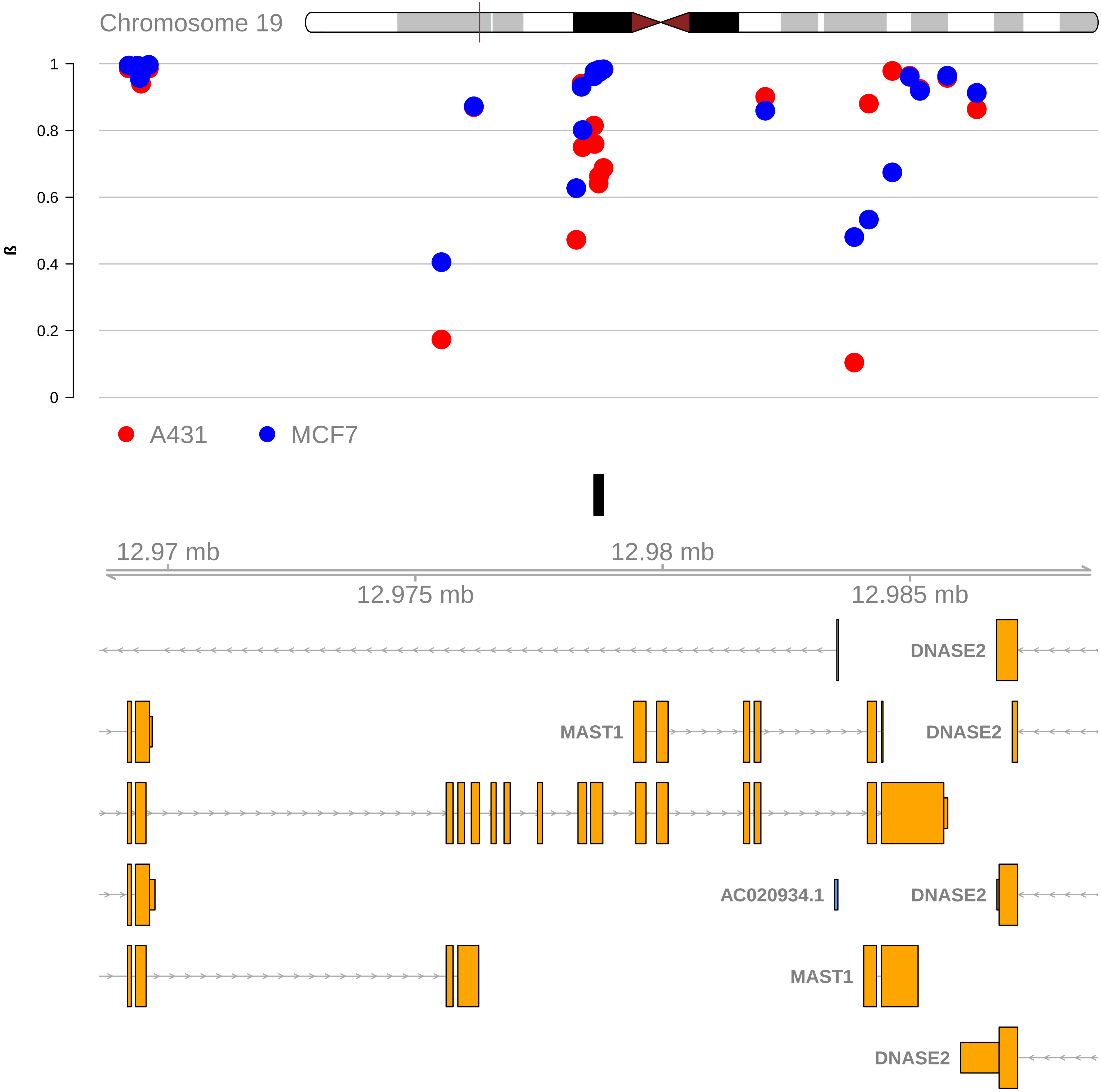

Chromosome 19
ß
1
0.8
0.6
0.4
0.2
0
A431
MCF7
12.97 mb
12.98 mb
12.975 mb
12.985 mb
DNASE2
MAST1
DNASE2
AC020934.1
DNASE2
MAST1
DNASE2
